# Impact of a “Diagonal” Intervention on Uptake of Sexual and Reproductive Health Services by Female Sex Workers in Mozambique: A Mixed-Methods Implementation Study

**DOI:** 10.3389/fpubh.2018.00109

**Published:** 2018-04-18

**Authors:** Yves Lafort, Faustino Lessitala, Malica Sofia Ismael de Melo, Sally Griffin, Matthew Chersich, Wim Delva

**Affiliations:** ^1^International Centre for Reproductive Health, Ghent University, Gent, Belgium; ^2^International Centre for Reproductive Health-Mozambique, Maputo, Mozambique; ^3^Faculty of Health Sciences, Wits Reproductive Health and HIV Institute, University of the Witwatersrand, Johannesburg, South Africa; ^4^The South African DST-NRF Centre of Excellence in Epidemiological Modelling and Analysis (SACEMA), University of Stellenbosch, Stellenbosch, South Africa; ^5^Center for Statistics, Hasselt University, Diepenbeek, Belgium; ^6^Rega Institute for Medical Research, KU Leuven, Leuven, Belgium

**Keywords:** care seeking, female sex workers, HIV, mixed methods, Mozambique, sexual and reproductive health, implementation research

## Abstract

**Background:**

Female sex workers (FSWs) have high risks for adverse sexual and reproductive health (SRH) outcomes, yet low access to services. Within an implementation research project enhancing uptake of SRH services by FSWs, we piloted a “diagonal” intervention, which combined strengthening of FSW-targeted services (vertical) with making public health facilities more FSW-friendly (horizontal), and tested its effect.

**Methods:**

The study applied a convergent parallel mixed-methods design to assess changes in access to SRH services. Results of structured interviews with FSWs pre-intervention (*N* = 311) and thereafter (*N* = 404) were compared with the findings of eight post-intervention focus group discussions (FGDs) with FSWs and two with FSW-peer educators (PEs).

**Results:**

Marked and statistically significant rises occurred in consistent condom use with all partners (55.3–67.7%), ever use of female condoms (37.9–54.5%), being tested for HIV in the past 6 months (56.0–76.6%), using contraception (84.5–95.4%), ever screened for cervical cancer (0.0–16.9%) and having ≥10 contacts with a PE in the past year (0.5–24.45%). Increases mostly resulted from FSW-targeted outreach, with no rise detected in utilization of public health facilities. FGD participants reported that some facilities had become more FSW-friendly, but barriers such as stock-outs, being asked for bribes and disrespectful treatment persisted.

**Conclusion:**

The combination of expanding FSW-targeted SRH services with improving access to the public health services resulted in an overall increased uptake of services, but almost exclusively because of the strengthened targeted (vertical) outreach services. Utilization of public SRH services had not yet increased and many barriers to access remained. Our diagonal approach was thus only successful in its vertical component. Improving access to the general health services remains nevertheless important and further research is needed how to reduce barriers. Ideally, the combination approach should be maintained and more successful approaches to increase utilization of public services should be explored.

## Introduction

Female sex workers (FSWs) in low-resource countries are among the groups who are most vulnerable to adverse sexual and reproductive health (SRH) outcomes. They are at increased risk for sexually transmitted infections (STIs), including HIV and HPV (1–3). Sex workers are for example 10 times more likely than adults in the general population to acquire HIV (4). In countries with medium and high HIV prevalence, the overall HIV prevalence among sex workers was 30.7% in 2012 (1). Unintended and unwanted pregnancies are much more common among FSWs than among women in the general population (5–7), and they are also more often victim of sexual and other types of violence (8).

However, access to the general SRH services is hampered by stigmatization and being treated with disrespect by both providers and other service users, practicing sex work away from their place of origin, resulting in lack of familiarity with locally available health services and sometimes illegal immigration status, and unsuitable opening hours (9, 10). Their vulnerability can be further exacerbated by poverty, criminalization and repeated human rights violations, low community cohesion, and heavy episodic drinking (11–13). Consequently, the overall use of SRH commodities and services by FSWs is often low (14). Because of the above, several initiatives have been taken, in various settings, to establish separate, parallel health services specifically targeting FSWs (15). These initiatives are however generally small-scale, limited in geographical range and time, and not sustained or scaled-up (16, 17).

**Figure 1:**
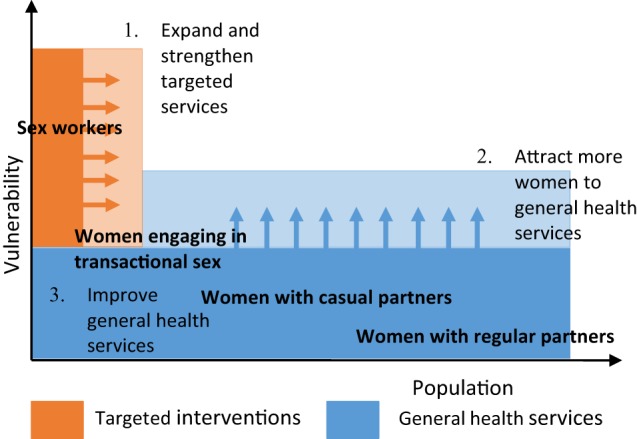
A “diagonal” approach to enhance access to health services for high-risk women.

Our hypothesis was that to ensure adequate access to SRH services, a “diagonal” approach is needed, combining FSW-targeted (vertical) interventions with facilitating access to the general (horizontal) health services, and establishing linkages between both (Figure 1). We piloted this approach in different settings and assessed its effect on SRH care seeking through a pre-post intervention comparison, using mixed-methods research. The current article presents the results of one of these settings: the Tete-Moatize area in Mozambique.

Studies testing the effect of different service-delivery approaches on care seeking behavior of FSWs are rare, and to our knowledge, our study is the first to compare FSW-targeted services with care seeking at public health facilities.

## Materials and Methods

### Contextual Background

The research was conducted in the context of a multi-country implementation research project, the Diagonal Interventions to Fast-Forward Reproductive Health (DIFFER) project (18). The project developed, piloted, and tested diagonal models to improve uptake of SRH services by FSWs in four different cities: Mysore, India; Mombasa, Kenya; Durban, South Africa; and Tete, Mozambique. At baseline, a comprehensive policy and situational analysis was done at each site and, based on these results, a site-specific intervention package was developed addressing the major gaps (19–21). In Mozambique, the study area comprised the adjoining urban areas of Tete and Moatize. The area has a total population of about 250,000 people. Sex work is common due to the presence of an important mining industry and major transport routes intersecting the area (22). Harassment of sex workers by law enforcers is common and more than half are originally from the neighboring countries, in particular Zimbabwe, and are often in the country illegally. Health services are available at eight public health centers, four private clinics, and one clinic specifically targeting FSWs and other key populations (the Moatize “Night Clinic”). This clinic is operated jointly by a non-governmental organization (NGO) and the district health department and offers information, condoms, STI treatment, family planning and HIV testing, as well as outreach peer education.

The baseline situational analysis demonstrated that the public sector was by far the main provider of SRH services in the area, with the private sector playing only a marginal role and the Night Clinic having limited coverage and scope of services (23, 24). The public SRH services were, however, not adapted to the needs of FSWs, faced serious limitations in terms of staff, space, equipment and supplies, and access by FSWs was hampered by them being asked for bribes, being poorly treated by some care providers, stigmatization and breaches of confidentiality. Gaps were noted in some services and commodities, such as the contraceptive implant, female condoms, cervical cancer screening and sexual and gender-based violence (SGBV) services. Termination of pregnancy (TOP) was illegal and therefore not officially offered. The use of SRH services and commodities by FSWs was overall insufficient and the coverage of the peer outreach limited (24).

The intervention package that was developed intended to address, where possible, the gaps detected and is described in detail elsewhere (25). In brief, the targeted component comprised the strengthening and expansion of the peer outreach activities, the provision of additional services at the Night Clinic, and clinical outreach. The number of peer educators (PEs), comprising both Mozambican and foreign FSWs, was increased, a micro-planning approach was adopted based on the Avahan model (26), the geographical range and scope of services was expanded, and additional training conducted. At the Night Clinic, lubricants, female condoms, contraceptive implants, emergency contraception and referral for cervical cancer screening were added to the scope of services.

Clinical outreach services, offering HIV testing and condoms, were conducted during 5 months by one NGO, and the same services, plus STI screening and contraception, were offered throughout the intervention period by another NGO.

To improve access to public services, a focal point for FSWs was appointed at four of the eight public health centers, who served as the contact person for FSW-related issues and regularly met with FSW representatives. In addition, the SRH care providers were trained in a FSW-friendly and FSW-adapted approach. Linkages were strengthened by establishing referral systems between the Night Clinic, outreach services and the public health services, and having regular joint meetings.

The intervention was gradually implemented from mid-2014 onward and evaluated during the first months of 2016. The evaluation assessed whether the intervention was feasible; acceptable to policy makers, health managers and providers; reasonable in cost and sustainable; effective in increasing uptake of services and acceptable to FSWs; and equitable. It applied a convergent parallel mixed-methods design (27, 28). The current article presents the results in relation to the effectiveness in increasing uptake and satisfaction by FSWs, which was evaluated through pre- and post-intervention cross-sectional surveys among FSWs, and post-intervention focus group discussions (FGDs) held with FSWs and with FSW PEs. The results of the other evaluation components (feasibility, acceptability, and sustainability) are available online (29).

### Cross-Sectional Surveys

Quantitative indicators of service uptake were measured at baseline and end-line through face-to-face interviews of a representative sample of FSWs recruited by respondent-driven sampling (30, 31). Both surveys were independent from each other and did not necessarily include the same FSWs. Procedures at baseline have been detailed elsewhere (18, 21, 29) and were identically repeated in the second survey. In brief, the project’s PEs identified eight “seeds” categorized according to relevant characteristics (nationality, place of residence, and number of clients). The seeds each invited three new FSWs from their social circle for the survey, using referral coupons, who in turn enrolled another three FSWs, and so on, until the desired sample was achieved (*N* = 400). Some of the recruitment chains died rapidly and an additional five seeds were therefore recruited. Referral coupons were managed using RDS Coupon Manager Version 3.0. Participants were interviewed face-to-face using an electronic questionnaire (QDS™, Version 3.0.1). The questionnaire assessed if the FSW had a need for different SRH commodities and services (condoms, contraception, STI care, HIV testing, HIV care, cervical cancer screening and sexual violence services), if the FSW had used the service, where the services had been obtained, satisfaction with the availability of services, and exposure to peer outreach. Interview data were stored in QDS Data Warehouse and merged with the coupon information.

Prevalence estimates and confidence intervals were calculated, adjusted for the unequal probability of inclusion due to varying social network sizes and the similarities in characteristics of persons within social networks using the Stata (Version 14.2, College Station, TX, USA) RDS analysis package. We used the “Volz-Heckathorn” estimator for calculating the weighted proportions and bootstrapping for the confidence intervals, correcting for non-normal distribution (30). We compared the uptake of different SRH care and prevention services between the two surveys, by merging the baseline and end-line datasets and assessing the results for statistically significant changes, fitting a logistic regression model with care seeking as dichotomous variable (1 = sought care, 0 = did not seek care), RDS-adjusted weights and using jack-knife resampling. We calculated a composite index, by adding in the denominator “1” for each service the FSW was in need of in the past year and in the numerator “1” for each of these services that the FSWs had used. FSWs with a score of 100% were considered as having used all the services they required. FSWs’ characteristics that were associated with uptake were stepwise introduced in the regression model and retained if they changed the odds ratio (OR) by at least 5%. Nationality and city of residence were included in all models. To assess if changes in uptake differed by FSWs’ characteristics, we included an interaction term in the model and calculated the adjusted ratios of the FSW characteristic-specific adjusted ORs.

For those who had sought care, we calculated prevalence estimates of where FSWs had most recently sought care and assessed for statistically significant changes, adjusting for nationality, city of residence and time residing in the area. To obtain an overall estimate, we created an additional database with each need for a care-seeking event for contraception, STI care or HIV testing as a separate observation (*N* = 1622) and we compared the place where care was sought, adjusting for the cluster effect of care-seeking need events by the same FSWs.

### Focus Group Discussions

Focus group discussions were held with FSWs of different characteristics: Two with Mozambican FSWs who only occasionally engaged in sex work, two with Mozambican FSWs for whom sex work was a full-time employment and two with foreign FSWs. To ensure uniformity in the latter group, only FSWs of Zimbabwean origin who engage full-time in sex work were included, since most foreign FSWs fall under this category (approximately 80% of foreign FSWs are Zimbabwean). Participants were recruited by the PEs and were at least 18 years old. In addition, two FGDs were held with FSW PEs, one with Mozambican FSW PEs and one with Zimbabwean FSW PEs. The number of participants per FGD ranged from five to nine. Using a semi-structured guide in English or Portuguese, a moderator explored where FSWs sought SRH services and why, perspectives toward the availability of SRH services, changes over the past 2 years, and persisting barriers to care. The topics discussed with the FSW PEs included their appreciation of the feasibility, adequacy, effectiveness, and sustainability of the peer outreach activities. The discussions were audio-recorded, transcribed, and analyzed using NVivo 11 (QSR International) independently by two researchers. The FSW transcripts were deductively and selectively coded (32), applying an axial matrix with in the *Y*-axis the themes (choice of place where SRH care sought, availability and quality of services, and barriers to access) and in the *X*-axis if the appreciation was positive or negative, and changes over the past 2 years. A similar analysis was done of the FSW PEs transcripts, but with the themes feasibility, adequacy, and efficacy in the *Y*-axis and a positive/negative appreciation in the *X*-axis.

### Mixed-Methods Analysis

A mixed analysis was carried out, applying a joint display strategy (27), arraying the results of the comparison of the pre- and post-intervention cross-sectional surveys, the FGDs with FSWs, and the FGDs with FSWs PEs, to formulate integrated conclusions on the following research questions: (1) did the use of HIV/SRH services and commodities by FSWs increase?; (2) did the place where FSWs seek care change and, (3) if so, why?; (4) are FSWs satisfied with the current availability of services?; and (5) what are the persisting barriers and gaps?

### Ethics

All study participants gave written informed consent; confidentiality was safeguarded through the use of non-identifying survey codes and storing information in password-protected computers. The study protocol was approved by the National Committee of Bioethics for Health in Mozambique (67/CNBS/2015).

## Results

### Participants’ Characteristics

Table 1 presents the characteristics of the participants of the two surveys. In the second survey, there were relatively more FSWs who resided in Tete City, had Mozambican nationality, were single, resided in the area for more than 3 years, and had a large number of clients. There were also differences in number of non-commercial partners (fewer in the second survey), proportion reporting HIV-positive status (lower), and proportion reporting to have been forced to have sex (higher).

**Table 1:** Characteristics of study participants in the pre- and post-intervention surveys.

Characteristics	First cross-sectional survey (*N* = 311)		Second cross-sectional survey (*N* = 404)	
		
	RDS-adjusted%	95% CI	RDS-adjusted%	95% CI
**Age (years)**				
Median	29		29	
≤20	16.2	8.6–23.7	17.6	11.8–23.9
21–25	20.6	15.0–26.2	25.6	19.4–32.4
26–30	26.9	19.8–34.0	28.9	22.6–35.7
31–35	19.6	14.2–24.9	14.3	10.1–19.0
≥36	16.7	11.3–22.1	13.6	9.1–18.9

**Place of residence**				
Moatize	51.9	43.8–59.1	31.5	26.0–38.1
Tete	48.1	40.9–56.2	68.5	62.5–74.3

**Nationality**				
Foreign	67.3	58.7–75.3	62.7	55.8–70.5
National	32.7	24.8–40.4	37.3	29.5–44.2

**Present relationship**				
Single, never married	32.6	25.6–39.9	26.8	20.8–33.3
Married or cohabiting	8.0	2.9–14.7	3.1	1.5–5.3
Single, previously married	59.4	51.6–67.2	70.0	63.5–76.3

**Years living in current residence**				
Median	2		3.7	
<3 years	54.7	47.0–62.3	45.9	38.9–53.2
≥3 years	45.3	37.7–53.0	54.1	46.8–61.1

**No of commercial sex acts in past week**				
Median	10		20	
<6	20.1	14.4–26.2	13.8	9.5–18.6
6–10	41.4	33.2–49.9	22.3	15.4–29.4
11–15	11.4	7.6–15.6	14.9	9.7–21.1
≥16	27.1	19.8–34.6	49.1	42.0–56.9

**Had a non-commercial partner in past month**				
Regular partner^a^	33.3	26.1–41.4	17.1	12.3–21.9
Occasional partner^a^	49.2	41.7–56.3	31.8	25.5–38.7

**Abnormal discharge or genital ulcer in past 12 months**				
Yes	49.5	42.2–57.3	48.4	41.3–55.7

**Result of last HIV test^b^**				
Positive	46.4	39.1–55.0	34.0	27.6–41.2

**Need for contraception^c^**				
Yes	91.2	86.5–95.3	97.3	95.2–99.0

**Forced to have sex in past 12 months**				
Yes	13.5	8.9–18.8	29.6	21.5–37.5

^a^A “regular” partner was defined as “a long-standing non-commercial partner who did not give you money or gifts in return for sex and toward whom you feel an emotional attachment” and an occasional partner as “those partners other than your regular partner(s) who did not give you money or gifts in return for sex.”

*^b^Female sex workers who never tested for HIV are excluded*.

*^c^Women who were not pregnant and not desiring pregnancy were defined as having a need for contraception*.

A total of 16 occasional Mozambican FSWs, 16 full-time Mozambican FSWs, and 14 Zimbabwean FSWs participated in the FGDs. Most participants were residing in Moatize, the median age was 26 years, the median number of years doing sex work in the Tete-Moatize area was three, and the median number of clients in the past week five. The sociodemographic characteristics of the FGD participants by type of participant are summarized in Table 2.

**Table 2:** Characteristics of focus group discussion (FGD) participants.

Characteristics	Occasional Mozambican female sex workers (FSWs) (*N* = 16)	Full-time Mozambican FSWs (*N* = 16)	Zimbabwean FSWs (*N* = 14)
Age (median)	30	24	34
Residing in Moatize	15	16	12
Residing in Tete	1	0	2
Years doing sex work in the area (median)	3	6	3
Number of clients in the past week (median)	5	7	4

The median age of the nine Mozambican and seven Zimbabwean PEs who participated in the FGDs was 34 years. Eleven were operating in the city of Tete and five in the city of Moatize.

### Changes in Uptake of SRH Services

Table 3 compares uptake of SRH commodities and services over time. Substantial and—after adjusting for relevant confounders—significant increases were observed in consistent condom use with all partners (55.3–67.7%), ever having used a female condom (37.9–54.5%), having tested for HIV in the past 6 months (56.0–76.6%), currently using a modern contraceptive method (hormonal, IUD, tubal ligation or condoms) (84.5–95.4%) and ever have been tested for cervical cancer (from 0–16.9%). We did not observe an increase in condom use at last sex with a client or care seeking for SGBV. We saw a substantial and significant increase in having used all HIV/SRH services needed (i.e., scoring 100% on the composite index), from 11.2% to 24.5%.

**Table 3:** Use of sexual and reproductive health commodities and services by female sex workers.

	First CSS		Second CSS		AOR	95% CI	*p*-Value
					
	*N*	%	*N*	%
**Condom use at last sex with**							
Any client	309	96.8	403	92.1	0.54	0.23–1.28	0.162
Regular non-commercial partner^a^	142	43.3	121	49.8	1.64	0.78–3.43	0.192
**Always uses condoms with all partners**							
Yes	311	55.3	404	67.7	1.99	1.22–3.23	0.006
**Ever used female condom**							
Yes	311	37.9	402	54.5	2.28	1.47–3.55	<0.001
**Care sought for last sexually transmitted infection (STI)/RTI syndrome^b^**							
Yes	172	80.0	208	87.8	1.98	0.76–5.17	0.160
**Tested for HIV in past 6 months^c^**							
Less than 6 months	214	56.0	316	76.6	3.16	1.72–5.83	<0.001
**Currently using HIV care services^d^**							
Yes	128	84.0	155	88.9	1.38	0.44–4.25	0.579
**Currently using modern contraception^e^**							
Yes	259	84.5	388	95.4	3.87	1.88–8.00	<0.001
**Currently using non-barrier modern contraception^e^**							
Yes	259	69.5	387	72.9	1.30	0.80–2.15	0.300
**Ever tested for cervical cancer**							
Yes	311	0.0	380	16.9	–	–	–
**Ever tested for cervical cancer (aged ≥30)^f^**							
Yes	147	0.0	177	25.2	–	–	–
**Sought medical care for last forced sex incident^g^**							
Yes	42	41.4^j^	109	37.3	0.92	0.32–2.66	0.879
**Used all HIV/sexual and reproductive health (SRH) services she needed**							
Yes	311	11.2	404	24.5	2.68	1.50–4.78	0.001
**Discloses that is a female sex worker (FSW) when visiting public health services**							
Yes	311	46.0	402	28.9	0.50	0.31–0.81	0.005
**Had contact with a FSW peer educator (PE) in last 12 months**							
Yes	311	20.7	397	29.1	2.82	1.58–5.04	<0.001
**Had at least 10 contacts with a FSW PE in last 12 months**							
Yes	311	0.5	403	24.4	122.1	23.9–626	<0.001
**Services or information received from PEs^h^**							
Condoms	131	56.7	233	94.9	21.4	6.27–73.0	<0.001
Clinic referral^i^	131	30.4	233	52.7	3.74	1.85–7.53	<0.001

*^a^Only FSWs who had a regular partner in the past month were included*.

*^b^Only FSWs who had abnormal discharge or genital ulcer in past 12 months were included*.

*^c^Only FSWs who did not test positive for HIV more than 6 months ago were included*.

*^d^Only FSWs whose last HIV result was positive were included*.

*^e^Only FSWs who were not pregnant and had no desire for pregnancy were included*.

*^f^Only FSWs years or more were included*.

*^g^Only FSWs who were forced to have sex in past 12 months were included*.

*^h^Only FSWs who had contact with a PE in the past 12 months were included*.

*^i^Referral for STI care, HIV testing or HIV care*.

*^j^Bootstrap analysis was not possible because of too few observations in some categories (unadjusted proportion presented)*.

*AOR, adjusted odds ratio*.

Significantly more FSWs reported to have had a contact with a FSW PE in the past 12 months than at baseline (20.7–29.1%) and the proportion that had at least 10 contacts increased from 0.5% to 24.4%. Those who had a contact received condoms more often (56.7–94.9%) and were more often referred for either STI care, HIV testing, or HIV care.

The increase in uptake of services, as measured by the composite index, differed between sub-populations (Table 4). It was substantially and significantly higher in FSWs who had Mozambican nationality (adjusted ratio of AOR = 6.74, *p* = 0.015), and in FSWs who were widowed or separated (adjusted ratio of AOR ratio = 3.62, *p* = 0.049).

**Table 4:** Changes in the proportion accessing all sexual and reproductive health services, by characteristics of female sex workers.

	First CSS	Second CSS	*N*	RDS%	AOR^a^	AOR ratio^b^	95% CI	*p*-Value
								
	*N*	RDS%						
**Age^c^**								
28 years or less	151	20.5	200	32.1	1.79	1.50	0.57–3.97	0.410
More than 28 years	160	24.9	204	33.1	2.04			

**Place of residence**								
Tete	158	11.2	231	18.4	1.70	2.09	0.67–6.54	0.206
Moatize	153	11.2	173	37.8	4.70			

**Nationality**								
Foreign	233	13.7	284	17.7	1.64	6.74	1.44–31.6	0.015
Mozambican	78	6.0	120	36.0	8.01			

**Present relationship**								
Married/cohabiting/single	184	13.9	298	21.2	1.66	3.62	1.00–13.05	0.049
Widowed/separated	127	7.2	106	32.2	4.25			

**Years living in current residence**								
<3 years	163	12.3	168	19.0	1.32	2.17	0.67–6.99	0.196
≥3 years	148	9.8	230	29.6	4.19			

**Was away for more than a month in past year**								
No	209	12.8	267	27.4	2.40	1.79	0.32–9.92	0.507
Yes	102	6.8	104	19.7	5.20			

**No of commercial sex acts in past week**								
More than 15	87	14.9	238	29.8	1.85	0.97	0.29–3.26	0.958
15 or less	224	10.0	166	19.5	3.23			

**Price per sex act**								
More than 2.5 EUR	158	8.8	155	17.1	2.48	1.42	0.43–4.65	0.564
2.5 EUR or less	153	13.6	248	30.7	2.49			

**Had a non-commercial regular partner in the past month**								
Yes	110	15.9	81	19.4	1.20	3.69	0.83–16.3	0.085
No	201	8.8	323	25.6	3.02			

*^a^Characteristic-specific adjusted odds ratio (OR)*.

*^b^Adjusted ratio of the characteristic-specific adjusted ORs*.

*^c^Because cervical cancer screening is, according the Mozambican guidelines, not expected to be done in women 28 years old or less, this service was excluded from the composite index in this comparison*.

Mozambican FGD participants overall agreed that the availability of HIV/SRH services had improved over the past years. Mozambican full-time FSWs stated that in particular the availability of male condoms and peer outreach improved, while Mozambican occasional FSWs remarked on the rise in availability of condoms and lubricants, but even more so for HIV testing and cervical cancer screening.

There are changes, because with the help of the peer educators, advice as well, from the community-advisors, there is a lot of change. (Mozambican full-time FSW)

Looking back, it improved in fact, when you go there they receive you with urgency. (Occasional Mozambican FSW)

Zimbabwean FSWs held different views. Some felt that the availability had improved, but others not. The PEs believed that their activities had a positive effect on the FSWs’ behavior, that almost all were now consistently using condoms with clients, and that the use of contraception had increased.

### Changes in Where FSWs Seek SRH Services

The results on where FSWs last obtained SRH commodities and services are presented in Table 5. We observed that, over the study, condoms were significantly more often obtained from PEs and organizations and less from the market or stalls, shops or supermarkets, public health facilities, and the Night Clinic. Compared to baseline, contraception, STI care, and HIV testing services were relatively less often procured at public health facilities post-intervention. Nevertheless, despite these declines, public facilities continued to be the most common source of SRH services. Also, over the course of the study, the percent using services at the Night Clinic was lower than at baseline, although that these decreases were not statistically significant.

**Table 5:** Where female sex workers (FSWs) obtained sexual and reproductive health (SRH) commodities and services.

	RDS adjusted%				*p*-Value
		
	*N* = Those who used the service^a^		AOR	95% CI
			
	First CSS	Second CSS			
**Condoms**	***N* = 310**	***N* = 403**			
Night clinic	36.0	18.0	0.56	0.32–0.96	0.035
Market/stalls	30.8	24.1	0.42	0.25–0.72	0.001
Public hospital/health center	22.8	11.8	0.39	0.22–0.81	0.001
Organizations	13.9	42.4	6.63	3.78–11.6	<0.001
Peer educators	11.3	56.2	11.8	7.20–19.4	<0.001
Pharmacies	5.8	(1.8)	0.34	0.10–1.14	0.081
Shops/supermarkets	5.5	1.1	0.20	0.05–0.77	0.020
Friends	2.6	8.8	3.25	0.76–13.9	0.112
Bars/nightclubs	1.9	1.3	0.45	0.04–5.19	0.522

**Contraception**	***N* = 178**	***N* = 278**			
Public hospital/health center	35.4	34.7	0.47	0.22–0.99	0.046
Night Clinic	32.3	20.7	1.06	0.49–2.28	0.876
Outside the Tete-Moatize area	13.7	25.9	1.87	0.96–4.27	0.063
Mobile clinics	3.2	8.3	2.62	0.79–8.67	0.114
Pharmacies	13.3	6.0	0.44	0.19–1.04	0.063

**Sexually transmitted infection (STI) care**	***N* = 134**	***N* = 189**			
Public hospital/health center	62.3	44.4	0.35	0.18–0.68	0.002
Night clinic	24.5	13.8	0.72	0.34–1.49	0.374
Outside the Tete-Moatize area	7.8	10.3	1.15	0.36–3.71	0.816
Mobile clinics	0.0	12.9	–	–	–
Informal sector	7.5	15.2	2.34	0.61–9.04	0.217

**HIV testing**	***N* = 241**	***N* = 293**			
Public hospital/health center	41.4	30.3	0.46	0.25–0.83	0.011
Night Clinic	17.5	14.6	1.21	0.56–2.63	0.627
Outside the Tete-Moatize area	17.1	17.3	0.94	0.46–1.92	0.872
Mobile clinics	23.7	34.4	1.69	0.97–2.94	0.065

**Contraception, STI care and HIV testing combined**	***N* = 523**	***N* = 760**			
Public hospital/health center	44.3	35.2	0.44	0.31–0.64	<0.001
Night Clinic	24.2	16.7	0.99	0.66–1.50	0.966
Outside the Tete-Moatize area	14.3	18.9	1.32	0.85–2.06	0.216
Mobile clinics	14.5	21.3	1.60	1.01–2.54	0.047

**HIV care**	***N* = 105**	***N* = 141**			
Public hospital/health center	60.5	48.3	0.63	0.29–1.36	0.238
Outside the Tete-Moatize area	39.4	52.3	1.78	0.85–3.77	0.127

**Cervical cancer screening**		***N* = 84**			
Public hospital/health center	–	25.2	–	–	–
Outside the Tete-Moatize area	–	74.8	–	–	–

*^a^Condoms: All FSWs were included; Contraception: Only FSWs who used a non-barrier contraceptive method were included; STI care: Only FSWs who sought care for last STI episode in the past year were included; HIV testing: Only FSWs who were tested for HIV in the past year were included; HIV care: Only FSWs who were in HIV care were included*.

*AOR, adjusted odds ratio*.

Marked rises occurred in obtaining contraception, STI care and HIV testing from mobile clinical services, and this was significant (*p* = 0.047) for all visits combined. Procuring services outside the Tete-Moatize area, mostly in the area where the FSWs were from, continued to be important, particularly for cervical cancer screening (74.8%), HIV care (52.7%) and contraception (25.9%).

Focus group discussion participants mentioned the same sources for SRH commodities and care reported by the cross-sectional survey participants: mostly public health facilities, the Night Clinic and outreach services, and sometimes pharmacies or the market. SGBV services were sought from community workers and at specific SGBV departments attached to some police stations. Mozambican occasional FSWs attributed the improved availability of HIV testing, condoms, and lubricants to the outreach.

In relation to the past. It isn’t the same because before these, these… [outreach health providers] we didn’t see them circulate. Only these days we always see them and they come with all you need and they give it to you. Before they didn’t come, they didn’t come, and you had to go to the hospital, explain everything, how you feel. (Occasional Mozambican FSW)

The Mozambican FGD participants mentioned cost as the most important reason for choosing the Night Clinic or public facilities, because the services are free, while one has to pay at pharmacies, the market or traditional healers. The second most important motivation for a particular service provider was proximity. Zimbabwean FSWs also mentioned cost and proximity, but for these women, being treated with respect and feeling secure were stronger influences on their choice of provider. These were given as a reason for procuring services in Zimbabwe, where they are less stigmatized.

Yes, she said “Ah, it’s better you get that in Zimbabwe.” I don’t want to say that in Zimbabwe we are not insulted, we are insulted as well, but it is better. (Zimbabwean FSW)

Some of the Zimbabwean FSWs mentioned that levels of health worker abuse had reduced since the FSW focal points were introduced at public health facilities.

It depends where you go. When you go where there is someone who represents the sex workers, they receive you well and you even do not have to stand in line. You arrive and they help you. (Zimbabwean FSW)

The PEs also noted that the appointment of focal points at selected health facilities was a very useful approach and said to have had a positive effect on accessing the services.

We have focal points to ensure that the sex workers are received in a secure manner, in a secure manner, that doesn’t mean that without these focal points they cannot be received well, but it is a way to have a “godfather” or “godmother.”

Nevertheless, the fact that not all providers had been trained in FSW-friendly services still hampered access, in the event that the focal point was not present. This appeared to be facility-specific, with some facilities not having this problem, and, concurring with what the FSWs said, non-Mozambican FSWs still appeared to suffer more from discrimination by some providers than Mozambicans.

She was received by a nurse who doesn’t know who we are. She hadn’t been trained by ICRH [the implementing NGO], and started to laugh. She took the register and started to laugh. She [the FSW] became demoralized and didn’t even go, didn’t go for a consultation.

### Satisfaction With Current Availability of Services

Table 6 shows the results on the responses given when women were asked, post-intervention, if they were satisfied with the availability of different SRH commodities and services. Satisfaction with the availability of male condoms and services for contraception, STI care, HIV testing, HIV care, and SGBV was very high (≥95%). It was also high, although a little less, for services when having an unwanted pregnancy (91%) and lowest for the availability of female condoms (74%) and lubricants (65%).

**Table 6:** Satisfaction with the availability of sexual and reproductive health (SRH) services.

Service	*N*	RDS adjusted%	95% CI
Finds male condoms sufficiently available	404	95.9	93.2–98.0
Finds female condoms sufficiently available^a^	384	73.5	67.0–80.0
Finds lubricants sufficiently available^b^	352	64.5	56.8–71.7
Is satisfied with the availability of unwanted pregnancy services^c^	32	90.6^g^	–
Is satisfied with the availability of contraceptive services	397	94.5	91.4–97.1
Is satisfied with the availability of sexually transmitted infection (STI) care services^d^	205	97.7	94.8–99.3
Is satisfied with the availability of HIV testing services	403	98.3^g^	–
Is satisfied with the availability of HIV care services^e^	141	97.3	93.5–99.0
Is satisfied with the availability of sexual and gender-based violence (SGBV) care services^f^	53	95.0	86.8–98.9

*^a^Only FSWs who knew what a female condom was were included*.

*^b^Only FSWs who knew what a lubricant was were included*.

*^c^Only FSWs who had an unwanted pregnancy in the past 5 years were included*.

*^d^Only FSWs who had an STI in the past 12 months were included*.

*^e^Only FSWs in HIV care were included*.

*^f^Only FSWs who were victim of forced sex in the past year and sought care were included*.

*^g^Bootstrap analysis was not possible because of too few observations in some categories. The unadjusted proportion is presented instead*.

Focus group discussion participants were also overall satisfied with the current availability and quality of the HIV/SRH services, even after probing. In particular the availability of male condoms was said to be very good.

### Persisting Barriers and Gaps

Barriers to access were explored in the FGDs. The most common barrier to access public health facilities or the Night Clinic mentioned was the frequent stock-outs of certain family planning methods and STI drugs, in which event they were bought at pharmacies or the market.

For example, some days ago I was bad with discharge. I went to the hospital, made an appointment, they said I needed Kanamycin and pills, those that you insert (in the vagina). I spoke to the doctor and he said “Here we do not have injections, but I have a friend who has, will you buy it?” (Mozambican occasional FSW)

The practice of public health providers asking for bribes persisted. This was in particular the case for obtaining TOP or post-abortion care. A common practice in TOP appeared to be to first seek medicines from traditional healers or at the market and then go to a health facility for post-abortion care (vacuum aspiration). However, high prices were often asked, also at the public health facilities.

It is available, yes. But when you go there after an abortion, to have a washing, you have to give money, at least a little bit, to be attended. They do not accept to do a “curettage” for free. (Mozambican occasional FSW)

Participants agreed that disrespectful treatment by public health providers had diminished, but still persisted, at least by some providers.

Because, years ago, it was enough to see that you are Zimbabwean, they wouldn’t attend you. It was only insults, contempt and they only insulted you, insulted you. But now, it is better. They can insult you, just to insult you, but they don’t exaggerate as before. They do with more fear. (Zimbabwean FSW)

Mozambican occasional FSWs said that verbal abuse was particularly prevalent when seeking care for STIs or SGBV, and they considered this as unavoidable.

I was sick, uh, instead of giving me advice, she only made it worse with insults. I didn’t want to have that disease. (Mozambican occasional FSW)

Zimbabwean FSWs complained more strongly about persisting ill treatment and said that often Mozambicans do receive treatment, but Zimbabwean FSWs do not. Disrespectful treatment was mentioned by them in particular for HIV treatment and SGBV services and by one provider in particular. They recommended that these services should be offered at the Night Clinic.

And then they said, tell her to come, and then I heard like this: “Come here you Zimbabwean, what did you come to do here? You came to destroy our country, didn’t you?” And you feel sick as patient, but what can you do! Then you think: I can only wait until they receive me, you want help. I would be better it they would help us. (Zimbabwean FSW)

The persisting fear of poor reception was also reflected in the second cross-sectional survey, where the percentage of FSWs reporting to disclose to be a FSW when visiting a public health service was low (28.9%), and even lower than at baseline (46.0%) (Table 2).

Lack of privacy when collecting medicines in public facilities, lack of information on where and when they should go for cervical cancer screening, the long waiting lines at public facilities, and delays in initiating HIV treatment were also mentioned as barriers by some participants.

The commodities or services that were said by the PEs to be most lacking and for which there was a high demand by the FSWs were lubricants and mobile clinical services.

We should have an ambulance, we should have equipment to do testing in the field, that work that we did with the mobile clinic, we should have done it in more areas, in more areas. (Mozambican PE)

They don’t manage to get lubricants, because those are rare. We only give condoms. We don’t give lubricants, but the people need them. (Zimbabwean PE)

They confirmed that many foreign FSWs were in HIV care in their country of origin and that problems arise when they run out of antiretroviral drugs, because at the Mozambican health facilities they do not know what regimen they are following.

## Discussion

We assessed the impact of an intervention that combined the strengthening of FSW-specific, targeted activities (vertical), both at a stand-alone clinic and through outreach, with facilitation of access to the general health services (horizontal), on the utilization of SRH commodities and services by FSWs. Comparison of results from the two cross-sectional surveys suggests that overall these efforts raised utilization. For condoms, contraception, and in particular STI care and HIV testing, this was mostly because of the FSW-targeted outreach activities conducted. The FGD results confirmed that outreach services were in high demand and greatly enhanced access.

Community- and home-based outreach has previously been shown to be effective in reaching underserved populations with both HIV testing and contraceptive services (33–36). Few studies have assessed the effect of clinical outreach specifically targeting sex workers in resource-limited settings, but those that did conclude that it allowed larger numbers to be reached (37, 38). Our study also suggests that it is the most effective way to rapidly increase uptake.

We did not detect an increase in service use at the Night Clinic, the other FSW-targeted, vertical service delivery component. A separate analysis of the clinic statistics showed only a slight rise in patient numbers (29). The most probable reason is that the originally planned expansion of the services could not be fully implemented. The construction of a second clinic in Tete City was canceled because of withdrawal of support by a private partner and the government, and permission from local authorities was not granted for some additional services to be offered at the clinic (cervical cancer screening, post-abortion care and initiation of HIV care). In addition, some of the FSWs reached by the outreach might otherwise have gone to the clinic.

The intervention did not result in an increase of the utilization of the SRH services at public health facilities either (the “horizontal” component of the intervention), except for cervical cancer screening. The increase in FSWs reporting to have been screened for cervical cancer (from 0% to 17%) is likely attributable to the fact that this period coincided with the roll out of screening services to all public health facilities during the intervention period.

Despite the appointment of focal points and provider training being well appreciated by the FGD participants, and said to have improved access, this did not yet translate into increased utilization, nor in women disclosing as FSWs when visiting public facilities. Barriers such as being asked for bribes and poor treatment by providers persisted, especially among Zimbabwean FSWs, although to a lesser degree than before. The activities to improve access to public health services started relatively late into the project and it is possible that at the time of the evaluation it was still too early to detect change. To our knowledge, no study has ever tested the impact of making public health services FSW-friendly and little is known about how to do so effectively.

The Mozambican Ministry of Health has recently adopted a strategy to make the public health services more key population-friendly and therefore no longer sees a need for parallel clinics targeting key populations, such as the Night Clinic (29). Targeted outreach offering peer education, condom distribution and HIV testing by NGOs is endorsed by the government, but not directly supported. FSW-targeted outreach is currently only offered by NGOs and is dependent on short-term external funding, posing challenges of sustainability. Our findings indicate that improving access to public health facilities is not easily achieved, and that further monitoring of the uptake of SRH services at public facilities, and evidence that uptake by FSWs is indeed improving, is needed before halting the targeted services. Sustainable funding needs to be ensured in the meantime.

Another persisting barrier that emerged from the FGDs are frequent stock-outs of STI treatment and contraceptive methods, both at the public health facilities and the Night Clinic. Fragile supply chains in the public sector are a reality in many resource-limited countries such as Mozambique (39, 40). To enhance sustainability, the Night Clinic commodities are supplied by the government and suffer the same stock-outs. The problem is thus not specific to FSWs, but affects all SRH care users.

An important finding is that a substantial proportion of the FSWs procured services outside the project area. These are mostly foreign FSWs who seek care at their place of origin. This poses challenges in ensuring uninterrupted care, in particular for HIV treatment. The link between mobility and poor retention in HIV care has been well documented but effective strategies to tackle the problem are still lacking (1, 41). This issue was insufficiently addressed by the intervention and there is a need to establish guidelines and procedures on this matter, and linkages with neighboring countries.

The intervention had a lesser effect on increasing care seeking by FSWs of foreign origin and this needs to be further explored. A partial explanation is that care seeking was already higher among foreign FSWs at baseline, but also that stigmatization of this group is higher and more difficult to reduce. Further sensitization and monitoring of health care providers behaviors toward foreigners is clearly required.

The coverage of the peer outreach, offering education, condoms and referrals, increased substantially, but remained insufficient. The available resources only allowed the project to employ 20 PEs, not enough to achieve adequate coverage. Peer outreach has repeatedly been shown to be effective and have the potential to reach full coverage, for example in India, but also in parts of Africa (19, 42, 43). The Mozambican government is developing a national strategy on peer outreach to key populations (29), but funding will remain highly dependent on short-term projects, with concomitant challenges to sustainability.

Female sex workers reported high satisfaction with the availability of most SRH commodities and services, both in the survey and FGDs. The only commodities that appear to have limited availability are lubricants and the female condom, because they are only distributed irregularly by the targeted services and not routinely within the public service. The lack of free post-abortion care was also mentioned. TOP and post-abortion care were the services said to be most lacking at baseline (24). Because TOP was illegal at the time, there was little that the project could do to improve access. However, TOP has recently been decriminalized in Mozambique (44) and during the roll-out of these services it will be especially important to ensure access of FSWs to these services.

Our study has limitations inherent in its design and these were mitigated as much as possible. Face-to-face interviews are challenged by recollection bias, poor understanding of the question, social desirability bias, and reluctance to divulge sensitive personal information (45). We minimized differential bias across the two surveys by phrasing the questions in the same way and using the same response options. We used RDS to ensure inclusion of hard-to-reach FSWs, but this relies on a successful recruitment of participants by their peers. Many of the initial seeds only recruited a few participants and had to be replaced by other seeds. We ensured however that the final recruitment chains branched into all FSW sub-populations. The refusal rate among Mozambican FSWs for the baseline survey was high (24) and this might be one of the reasons behind the differences in FSWs’ characteristics between the two surveys. We adjusted for these differences in our logistic regression analysis, thereby reducing a possible confounding effect. FGDs allow a more in-depth exploration of the responses and facilitate a more natural discussion, but the representativeness of the participants is not assured and responses can be driven by the more outspoken participants (46). These limitations were mitigated by our mixed-methods approach. Both methods are complementary and the comparison of the results of the cross-sectional surveys with those of the FGDs permitted integrated and valid conclusions.

Finally, our study was a pre-post comparison without control. Each FSW setting is specific and it was not possible to identify another setting that was sufficiently similar to serve as control. Pre-post intervention studies are increasingly being accepted as a valid design for implementation research studies (47, 48). Their strength is that they demonstrate the effect of an intervention in a real-life situation. The disadvantage is that we cannot assign the measured effect with complete certainty to the piloted intervention. Nevertheless, no other interventions took place during the study period and the analysis of where FSWs sought care provided sufficient evidence to attribute increases in care seeking to certain intervention components.

## Conclusion

A “diagonal” intervention, combining the expansion of vertical FSW-targeted SRH services with improving access horizontally to the public health services, was successful in increasing uptake of services by FSWs, although almost exclusively because of the “vertical” component of targeted outreach conducted. Utilization of public SRH services did not (yet) show any increase, with important barriers persisting. Our diagonal approach was thus only successful in its vertical component. Nevertheless, improving access to the general health services remains important. Close monitoring, to assess impact over a longer period of time, together with additional strategies to optimize the horizontal component of our approach are needed.

## Ethics Statement

All study participants gave written informed consent and received an information sheet, confidentiality was safeguarded through the use of non-identifying survey codes and storing information in password-protected computers. The study protocol was approved by the National Committee of Bioethics for Health in Mozambique (67/CNBS/2015).

## Author Contributions

YL is the coordinator of the DIFFER project and the principal investigator of the study in Mozambique. He had the lead in the development of the study protocol and the data collection tools, and adapted them to the Mozambican context. He analyzed all the collected information and had the lead in the writing of the study reports and of the article. FL coordinated the data collection in Mozambique, provided feedback to the analysis and contributed to the writing of the study report and the article. MSIM analyzed the focus group discussions conjointly with YL, and contributed to the writing of the study report and the article. SG is the coordinator of the DIFFER project in Mozambique, contributed to the development of the study protocol and data collection tools, and contributed to the writing of the study report and the article. MC was a principal investigator of the DIFFER project in South Africa and provided inputs to the development of the study protocol and data collection tools, and contributed to the writing of the article. WD is the PhD promotor of the first author, provided feedback on the study design, oversaw the analysis of the study results and the writing of the article.

## Conflict of Interest Statement

The authors declare that the research was conducted in the absence of any commercial or financial relationships that could be construed as a potential conflict of interest.

## References

[1] Baral S Beyrer C Muessig K Poteat T Wirtz AL Decker MR Burden of HIV among female sex workers in low-income and middle-income countries: a systematic review and meta-analysis. Lancet Infect Dis (2012) 12(7):538–49. 10.1016/S1473-3099(12)70066-X 22424777

[2] Pruss-Ustun A Wolf J Driscoll T Degenhardt L Neira M Calleja JMG. HIV due to female sex work: regional and global estimates. PLoS One (2013) 8(5):e63476. 10.1371/journal.pone.0063476 23717432 PMC3662690

[3] Cwikel JG Lazer T Press F Lazer S. Sexually transmissible infections among female sex workers: an international review with an emphasis on hard-to-access populations. Sex Health (2008) 5(1):9–16. 10.1071/SH07024 18361849

[4] UNAIDS. Prevention Gap Report. Geneva: UNAIDS (2016).

[5] Morineau G Neilsen G Heng S Phimpachan C Mustikawati DE. Falling through the cracks: contraceptive needs of female sex workers in Cambodia and Laos. Contraception (2011) 84(2):194–8. 10.1016/j.contraception.2010.11.003 21757062

[6] Schwartz S Papworth E Thiam-Niangoin M Abo K Drame F Diouf D An urgent need for integration of family planning services into HIV care: the high burden of unplanned pregnancy, termination of pregnancy, and limited contraception use among female sex workers in Cote d’Ivoire. J Acquir Immune Defic Syndr (2015) 68:S91–8. 10.1097/QAI.0000000000000448 25723996

[7] Sutherland EG Alaii J Tsui S Luchters S Okal J King’ola N Contraceptive needs of female sex workers in Kenya – a cross-sectional study. Eur J Contracept Reprod Health Care (2011) 16(3):173–82. 10.3109/13625187.2011.564683 21413869

[8] Decker MR Crago AL Chu SKH Sherman SG Seshu MS Buthelezi K Human rights violations against sex workers: burden and effect on HIV. Lancet (2015) 385(9963):186–99. 10.1016/S0140-6736(14)60800-X 25059943 PMC4454473

[9] Scorgie F Nakato D Harper E Richter M Maseko S Nare P ’We are despised in the hospitals’: sex workers’ experiences of accessing health care in four African countries. Cult Health Sex (2013) 15(4):450–65. 10.1080/13691058.2012.763187 23414116

[10] Vuylsteke B Ghys PD Mah-bi G Konan Y Traore M Wiktor SZ Where do sex workers go for health care? A community based study in Abidjan, Cote d’Ivoire. Sex Trans Infect (2001) 77(5):351–2. 10.1136/sti.77.5.351 PMC1744355 11588281

[11] Moore L Chersich MF Steen R Reza-Paul S Dhana A Vuylsteke B Community empowerment and involvement of female sex workers in targeted sexual and reproductive health interventions in Africa: a systematic review. Global Health (2014) 10:47. 10.1186/1744-8603-10-47 24916108 PMC4074148

[12] Scorgie F Chersich MF Ntaganira I Gerbase A Lule F Lo YR. Socio-demographic characteristics and behavioral risk factors of female sex workers in Sub-Saharan Africa: a systematic review. AIDS Behav (2012) 16(4):920–33. 10.1007/s10461-011-9985-z 21750918

[13] Scorgie F Vasey K Harper E Richter M Nare P Maseko S Human rights abuses and collective resilience among sex workers in four African countries: a qualitative study. Global Health (2013) 9:33. 10.1186/1744-8603-9-33 23889941 PMC3750273

[14] Mountain E Mishra S Vickerman P Pickles M Gilks C Boily MC. Antiretroviral therapy uptake, attrition, adherence and outcomes among HIV-infected female sex workers: a systematic review and meta-analysis. PLoS One (2014) 9(9):e105645. 10.1371/journal.pone.0105645 25265158 PMC4179256

[15] Awungafac G Delvaux T Vuylsteke B Systematic review of sex work interventions in sub-Saharan Africa: examining combination prevention approaches. Trop Med Int Health (2017) 22(8):971–93. 10.1111/tmi.12890 28449198

[16] Dhana A Luchters S Moore L Lafort Y Roy A Scorgie F Systematic review of facility-based sexual and reproductive health services for female sex workers in Africa. Global Health (2014) 10:46. 10.1186/1744-8603-10-46 24916010 PMC4070634

[17] Wilson D. HIV programs for sex workers: lessons and challenges for developing and delivering programs. PLoS Med (2015) 12(6):e1001808. 10.1371/journal.pmed.1001808 26079267 PMC4469316

[18] ICRH. The DIFFER Project: Home Page of the DIFFER Website (2011). Available from: http://differproject.eu/ (Accessed: December 22, 2015).

[19] Lafort Y Greener R Roy A Greener L Ombidi W Lessitala F HIV prevention and care-seeking behaviour among female sex workers in four cities in India, Kenya, Mozambique and South Africa. Trop Med Int Health (2016) 21(10):1293–303. 10.1111/tmi.12761 27479236

[20] Lafort Y Greener R Roy A Greener L Ombidi W Lessitala F Where do female sex workers seek HIV and reproductive health care and what motivates these choices? A survey in 4 cities in India, Kenya, Mozambique and South Africa. PLoS One (2016) 11(8):e0160730. 10.1371/journal.pone.0160730 27494412 PMC4975460

[21] Lafort Y Greener R Roy A Greener L Ombidi W Lessitala F Sexual and reproductive health services utilization by female sex workers is context-specific: results from a cross-sectional survey in India, Kenya, Mozambique and South Africa. Reprod Health (2017) 14:13. 10.1186/s12978-017-0277-6 28103896 PMC5247811

[22] Lafort Y Geelhoed D Cumba L Lazaro CDM Delva W Luchters S Reproductive health services for populations at high risk of HIV: performance of a night clinic in Tete province, Mozambique. BMC Health Serv Res (2010) 10:144. 10.1186/1472-6963-10-144 20507644 PMC2890643

[23] Lafort Y Jocitala O Candrinho B Greener L Beksinska M Smit JA Are HIV and reproductive health services adapted to the needs of female sex workers? Results of a policy and situational analysis in Tete, Mozambique. BMC Health Serv Res (2016) 16:301. 10.1186/s12913-016-1551-y 27456516 PMC4960856

[24] Lafort Y Lessitala F Candrinho B Greener L Greener R Beksinska M Barriers to HIV and sexual and reproductive health care for female sex workers in Tete, Mozambique: results from a cross-sectional survey and focus group discussions. BMC Public Health (2016) 16:608. 10.1186/s12889-016-3305-5 27440108 PMC4955167

[25] The DIFFER Consortium. Implementation of the Interventions: Final Report. Gent (2016). Available from: http://differproject.eu/Project_Documents (Accessed: April 2017).

[26] Foundation BMG. Peer Led Outreach at Scale: A Guide to Implementation. New Delhi: Foundation BMG (2009).

[27] Creswell JW Plano Clark VL Designing and Conducting Mixed Methods Research. 2nd ed Thousand Oaks: SAGE (2011).

[28] Curry LA Krumholz HM O’Cathain A Clark VLP Cherlin E Bradley EH Mixed methods in biomedical and health services research. Circ Cardiovasc Qual Outcomes (2013) 6(1):119–23. 10.1161/CIRCOUTCOMES.112.967885 23322807 PMC3569711

[29] The DIFFER Consortium. Evaluation of the Performance of the Implemented Differ Interventions: Final Report. Gent (2017). Available from: http://differproject.eu/Project_Documents (Accessed: April 2017).

[30] Salganik MJ Heckathorn DD Sampling and estimation in hidden populations using respondent-driven sampling. Sociol Methodol (2004) 34:193–239. 10.1111/j.0081-1750.2004.00152.x

[31] Johnston L Sabin K Sampling hard-to-reach populations with respondent driven sampling. Method Innov Online (2010) 5(2):38–48. 10.4256/mio.2010.0017

[32] Denzin NK Lincoln YS The SAGE Handbook of Qualitative Research. 5th ed Los Angeles: SAGE Publisher (2018). 968 p.

[33] Sharma M Ying R Tarr G Barnabas R. Systematic review and meta-analysis of community and facility-based HIV testing to address linkage to care gaps in sub-Saharan Africa. Nature (2015) 528(7580):S77–85. 10.1038/nature16044 26633769 PMC4778960

[34] Sweat M Morin S Celentano D Mulawa M Singh B Mbwambo J Community-based intervention to increase HIV testing and case detection in people aged 16-32 years in Tanzania, Zimbabwe, and Thailand (NIMH Project Accept, HPTN 043): a randomised study. Lancet Infect Dis (2011) 11(7):525–32. 10.1016/S1473-3099(11)70060-3 21546309 PMC3156626

[35] WHO. Service Delivery Approaches to HIV Testing and Counselling (HTC): A Strategic HTC Policy Framework. Geneva: WHO (2012).

[36] Scott VK Gottschalk LB Wright KQ Twose C Bohren MA Schmitt ME Community health workers’ provision of family planning services in low- and middle-income countries: a systematic review of effectiveness. Stud Fam Plann (2015) 46(3):241–61. 10.1111/j.1728-4465.2015.00028.x 26347089

[37] Dugas M Bedard E Batona G Kpatchavi AC Guedou FA Dube E Outreach strategies for the promotion of HIV testing and care: closing the gap between health services and female sex workers in Benin. J Acquir Immune Defic Syndr (2015) 68:S198–205. 10.1097/QAI.0000000000000463 25723985

[38] Mulongo S Kapila G Hatton T Canagasabey D Arney J Kazadi T Applying innovative approaches for reaching men who have sex with men and female sex workers in the democratic Republic of Congo. J Acquir Immune Defic Syndr (2015) 68:S248–51. 10.1097/QAI.0000000000000449 25723991

[39] International Health Partnership (IHP+). Joint Assessment of the Mozambican Health Sector Strategic Plan (PESS, 2014-2019): Final Report. Maputo: (2013).

[40] República de Moçambique, Ministério de Saúde. Relatório da Revisão do Sector de Saúde. Maputo: (2012).

[41] Beyrer C Baral S Kerrigan D El-Bassel N Bekker LG Celentano DD. Expanding the space: inclusion of most-at-risk populations in HIV prevention, treatment, and care services. J Acquir Immune Defic Syndr (2011) 57:S96–9. 10.1097/QAI.0b013e31821db944 21857306 PMC3164959

[42] Luchters S Chersich MF Rinyiru A Barasa MS King’ola N Mandaliya K Impact of five years of peer-mediated interventions on sexual behavior and sexually transmitted infections among female sex workers in Mombasa, Kenya. BMC Public Health (2008) 8:143. 10.1186/1471-2458-8-143 18445258 PMC2397398

[43] Laga M Galavotti C Sundaramon S Moodie R The importance of sex-worker interventions: the case of Avahan in India. Sex Transm Infect (2010) 86:I6–7. 10.1136/sti.2009.039255 20167733 PMC3252605

[44] REPÚBLICA DE MOÇAMBIQUE. BOLETIM DA REPÚBLICA. I SÉRIE — Número 105. 14.° SUPLEMENTO. Maputo: (2014).

[45] Langhaug LF Sherr L Cowan FM. How to improve the validity of sexual behaviour reporting: systematic review of questionnaire delivery modes in developing countries. Trop Med Int Health (2010) 15(3):362–81. 10.1111/j.1365-3156.2009.02464.x 20409291 PMC3321435

[46] Smithson J Using and analysing focus groups: limitations and possibilities. Int J Soc Res Methodol (2000) 3(2):113–9. 10.1080/136455700405172

[47] Peters DH Tran NT Adam T Implementation Research in Health: A Practical Guide. Geneva: World Health Organization (2013).

[48] Proctor E Silmere H Raghavan R Hovmand P Aarons G Bunger A Outcomes for implementation research: conceptual distinctions, measurement challenges, and research agenda. Adm Policy Ment Health (2011) 38(2):65–76. 10.1007/s10488-010-0319-7 20957426 PMC3068522

